# Proatherogenic Oxidized Low-Density Lipoprotein/**β**2-Glycoprotein I Complexes in Arterial and Venous Disease

**DOI:** 10.1155/2014/234316

**Published:** 2014-10-28

**Authors:** Jeffrey S. Berger, Caron B. Rockman, Kirk E. Guyer, Luis R. Lopez

**Affiliations:** ^1^Department of Medicine, Divisions of Cardiology and Hematology, New York University School of Medicine, New York, NY 10016, USA; ^2^Department of Surgery, Division of Vascular Surgery, New York University School of Medicine, New York, NY 10016, USA; ^3^Department of Chemistry, Indiana University, South Bend, IN 46634, USA; ^4^Medical Department, Corgenix, Inc., 11575 Main Street, No. 400, Broomfield, CO 80020, USA

## Abstract

OxLDL/*β*2GPI complexes have been implicated in the initiation and progression of atherosclerosis and associated with disease severity and adverse outcomes. We investigate the significance of anti-oxLDL/*β*2GPI antibodies and oxLDL/*β*2GPI complexes in patients with arterial and idiopathic venous disease. A cohort of 61 arterial disease patients, 32 idiopathic venous disease patients, and 53 healthy controls was studied. Because statins influence oxLDL/*β*2GPI, these complexes were analyzed on subjects not taking statins. Arterial and venous groups expressed higher levels of IgG anti-oxLDL/*β*2GPI antibodies than controls without any other significant clinical association. OxLDL/*β*2GPI complexes were significantly elevated in arterial (0.69 U/mL, *P* = 0.004) and venous disease (0.54 U/mL, *P* = 0.025) than controls (0.39 U/mL). Among arterial diseases, oxLDL/*β*2GPI was 0.85 U/mL for carotid artery disease, 0.72 U/mL for peripheral artery disease, and 0.52 U/mL for abdominal aortic aneurysm. There was a significant association with male gender, age, hypertension, and history of thrombosis. Subjects with oxLDL/*β*2GPI above the median (0.25 U/mL) were more likely to have arterial (OR 4.5, *P* = 0.004) or venous disease (OR 4.1, *P* = 0.008). Multivariate regression indicated that males (*P* = 0.021), high cholesterol (*P* = 0.011), and carotid disease (*P* = 0.023) were significant predictors of oxLDL/*β*2GPI. The coexistence of oxLDL/*β*2GPI in arterial and venous disease may suggest a common oxidative mechanism that independently predicts carotid artery disease.

## 1. Introduction

Atherosclerosis is a progressive disease of the arterial wall associated with an underlying systemic immune-inflammatory background that promotes dyslipidemia and arterial prothrombotic phenotypes. The oxidative modification of low-density lipoprotein (oxLDL) is one of the key events in the initiation and progression of atherosclerotic lesions [1]. OxLDL is highly inflammatory and immunogenically capable of further promoting endothelial dysfunction and enhanced secretion of chemotactic and proinflammatory cytokines including a proatherogenic immune response [2]. OxLDL interacts with the lipid binding plasma protein *β*2-glycoprotein I (*β*2GPI) to form oxLDL/*β*2GPI complexes [3], implicated as proatherothrombotic autoantigens [4, 5]. Further, the coexistence of oxLDL/*β*2GPI complexes and autoantibodies to these complexes in systemic lupus erythematosus (SLE) and antiphospholipid syndrome patients suggested a pathogenic role in both venous and arterial thromboembolic events [6, 7].

In some nonautoimmune patients positive for antiphospholipid antibodies and suffering from acute coronary syndromes, anti-*β*2GPI and anti-oxLDL/*β*2GPI antibodies were most prevalent [8]. In addition, circulating oxLDL/*β*2GPI complexes in these patients were associated with disease severity and with a 3.5-fold risk of 2-year adverse cardiovascular outcomes [9]. A recent report clearly demonstrated that rosuvastatin significantly reduced serum levels of oxLDL/*β*2GPI complexes in patients with diabetes, an effect that was independent of LDL levels but likely dependent on an antioxidant inhibition of nitric oxide metabolites [10]. The presence and significance of anti-oxLDL/*β*2GPI antibodies and oxLDL/*β*2GPI complexes have not been evaluated in nonautoimmune subjects with venous disease.

## 2. Methods

### 2.1. Patient Samples

We enrolled 146 subjects from the vascular surgery outpatient clinic at a tertiary medical center as follows: 61 with arterial disease, 32 with idiopathic venous disease, and 53 healthy controls. Since statins are known to lower oxLDL/*β*2GPI, we excluded subjects taking statins [10]. The demographic and clinical characteristics of 100 subjects not taking statins included in the study (27 arterial disease, 25 IVD, and 48 healthy controls) are summarized in Table 1. Of the 27 patients with arterial disease, 7 had carotid artery disease (CarAD), 11 peripheral artery disease (PAD), and 9 abdominal aortic aneurysm (AAA). Serum samples were obtained at study entry and were blind tested for IgG anti-oxLDL/*β*2GPI antibodies and oxLDL/*β*2GPI complexes.

### 2.2. ELISA for IgG Antibodies to oxLDL/*β*2GPI Complexes

IgG anti-oxLDL/*β*2GPI antibodies were measured by commercial ELISA (Corgenix, Inc., Broomfield, Colorado) following the manufacturer instructions. Diluted serum samples were incubated in microwells with immobilized oxLDL/*β*2GPI antigen complex to capture anti-oxLDL/*β*2GPI antibodies. Horseradish peroxidase (HRP) conjugated anti-human IgG antibodies were added to detect bound IgG anti-oxLDL/*β*2GPI antibodies followed by tetramethylbenzidine/H_2_O_2_ (TMB) as chromogenic substrate. Color development was stopped with 0.36 N sulphuric acid and optical density (OD) read at 450 nm (640 nm reference). IgG anti-oxLDL/*β*2GPI antibody concentration was calculated against a reference curve and results were expressed in G units.

### 2.3. ELISA for oxLDL/*β*2GPI Complexes

Serum oxLDL/*β*2GPI complexes were measured as previously described [10]. Briefly, IgG2b murine monoclonal antibody (3H3) specific for human *β*2GPI was coated onto 96-microwell plates and used to capture oxLDL/*β*2GPI complexes via its reactivity with *β*2GPI. Diluted patient serum was added to the appropriate microwells for incubation at room temperature for 1 hour. After washing, biotinylated 2E10 antibody (IgG murine monoclonal anti-human Apo B-100) was added to the microwells and incubated for 30 minutes, followed by Streptavidin-HRP for 30 minutes. Color was developed with tetramethylbenzidine/H_2_O_2_ (TMB) for 30 minutes and the reaction stopped with 0.36 N sulphuric acid. Optical density (OD) was read at a wavelength of 450 nm (650 nm reference). Serum oxLDL/*β*2GPI complex concentration (expressed in U/mL) was calculated against a reference curve built with threefold serial dilutions of a reference serum.

### 2.4. Statistical Analysis

For categorical variables, Chi-squared test or Fisher's exact test was used; for continuous variables paired *t*-test and Wilcoxon Signed Rank Sum test (when applicable) were used. Association between variables was assessed by univariate analysis (Pearson's rho) and the assumptions of the univariate analysis were tested by multiple regression. Cochran-Armitage Trend test was used to determine the trend between oxLDL/*β*2GPI quartiles and odds ratio (OR). Statistical analysis was performed using the JMP 9.0.0 program from SAS Institute Inc., NC, USA.

## 3. Results

The mean IgG anti-oxLDL/*β*2GPI antibody level for the arterial group was 15.3 ± 6.1 G units with 15.4% positive (above cut-off of the assay established at 20 G units) and 14.9 ± 4.3 G units with 12.1% positives for the venous group. These levels were not statistically different when compared to the healthy controls (13.4 ± 4.7 G units and 7.5% positives). Interestingly, the mean of the arterial and venous disease groups combined (15.2 ± 5.5 G units) was statistically higher than the controls (*P* = 0.037). No other association of IgG anti-oxLDL/*β*2GPI antibodies with demographic, serologic, or clinical variables was found.

**Figure 1:**
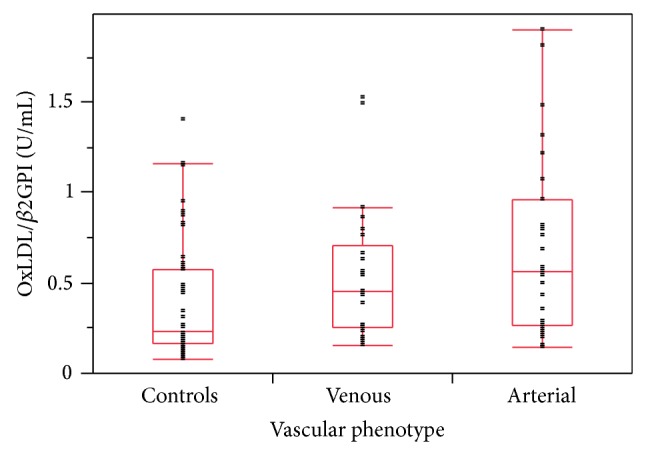
Distribution of serum levels of oxLDL/*β*2GPI complexes measured by ELISA in 48 healthy controls and 25 patients with venous and 27 with arterial diseases (*n* = 27). Subjects taking statins were excluded. Mean oxLDL/*β*2GPI levels in arterial (0.69 ± 0.50 U/mL, *P* = 0.004) and venous diseases (0.54 ± 0.37 U/mL, *P* = 0.025) were significantly higher than healthy controls (0.39 ± 0.33 U/mL). Mean levels in arterial (0.69 ± 0.50 U/mL) versus venous diseases (0.54 ± 0.37 U/mL) were not statistically different (*P* = 0.394). OxLDL/*β*2GPI levels among the three groups were statistically significant (*P* = 0.006, Wilcoxon/Kruskal-Wallis Rank Sum test). Boxes represent 75/25 percentiles with the horizontal line the median for the group and whiskers the 90/10 percentile bars.

**Figure 2:**
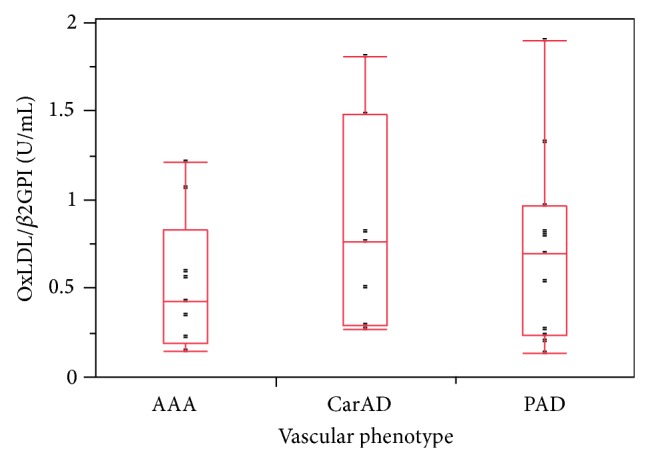
Distribution of serum levels of oxLDL/*β*2GPI complexes measured in 27 patients with arterial disease. Mean oxLDL/*β*2GPI levels of CarAD (0.85 ± 0.59 U/mL, *P* = 0.013) and PAD (0.72 ± 0.54 U/mL, *P* = 0.033) were significantly higher compared to healthy controls (0.38 ± 0.33 U/mL), while AAA (0.52 ± 0.38 U/mL, *P* = 0.255) were not. The trend among the arterial disease groups was not statistically significant (*P* = 0.466, Wilcoxon/Kruskal-Wallis Rank Sum test). Boxes represent 75/25 percentiles with the horizontal line the median for the group and whiskers the 90/10 percentile bars.

The mean oxLDL/*β*2GPI complex level of 100 subjects not taking statins was significantly elevated in arterial (0.69 ± 0.50 U/mL, *P* = 0.004) and venous groups (0.54 ± 0.37 U/mL, *P* = 0.025) compared to healthy controls (0.39 ± 0.33 U/mL) (Figure 1), and oxLDL/*β*2GPI levels between the arterial and venous disease groups were not different (*P* = 0.394). However, the association between the three groups was statistically significant (*P* = 0.006). Among patients with arterial disease, mean oxLDL/*β*2GPI levels for CarAD (0.85 ± 0.59 U/mL, *P* = 0.013) and PAD (0.72 ± 0.54 U/mL, *P* = 0.033) were significantly higher than healthy controls, but AAA (0.52 ± 0.38 U/mL, *P* = 0.255) was not. The association between the arterial disease subgroups was not statistically significant (*P* = 0.466) (Figure 2).

Subjects with oxLDL/*β*2GPI in the upper quartiles (above control median of 0.25 U/mL) were more likely to have arterial (OR 4.5, 1.54–13.14, *P* = 0.004) and/or venous diseases (OR 4.1, 1.38–11.99, *P* = 0.008) (Table 2). In unadjusted analysis, there was a significant association between oxLDL/*β*2GPI with male gender (0.55 versus 0.26 U/mL, *P* = 0.005), age (*r* = 0.299, *P* = 0.002), hypertension (0.54 versus 0.27 U/mL, *P* = 0.024), and history of thrombotic disease (0.95 versus 0.37 U/mL, *P* = 0.035). BMI was not associated with oxLDL/*β*2GPI levels. Multivariate regression models with oxLDL/*β*2GPI as a dependent variable indicated that male gender (*t* = 2.34, *P* = 0.021), high cholesterol (*t* = −2.58, *P* = 0.011), and CarAD phenotype (*t* = 2.32, *P* = 0.023) were significant predictors of oxLDL/*β*2GPI levels. Excluding venous disease, CarAD remained the only significant independent predictor of oxLDL/*β*2GPI levels (*t* = 2.30, *P* = 0.024). These results indicate a stronger proatherogenic role of oxLDL/*β*2GPI complexes in CarAD over the other arterial and venous vascular phenotypes studied.

## 4. Discussion

IgG anti-oxLDL/*β*2GPI antibodies in patients with arterial and venous diseases were higher than controls. This is consistent with current concepts for immune participation in atherothrombotic vascular disease. The relatively low prevalence and weak association of IgG anti-oxLDL/*β*2GPI antibodies with vascular phenotypes in this nonautoimmune patient population appear consistent with previous reports [8]. These results demonstrate significantly higher oxLDL/*β*2GPI complex levels in patients with arterial and venous diseases. This is the first report of high oxLDL/*β*2GPI in venous disease. In addition to statins, the clinical determinants that influenced serum levels of oxLDL/*β*2GPI were male gender, hypercholesterolemia, and carotid artery disease phenotype. Patients with oxLDL/*β*2GPI in the upper quartiles were equally more likely to have arterial (OR 4.5) and/or venous diseases (OR 4.1). The arterial disease results were consistent with a previous report that oxLDL/*β*2GPI complexes were associated with more severe coronary disease and poor outcomes [9]. Because patients with venous disease exhibited high oxLDL/*β*2GPI levels we hypothesize that arterial and venous thrombotic diseases share a similar underlying oxidative inflammatory mechanism. In arterial disease, lipid (LDL) peroxidation and oxLDL/*β*2GPI complexes promote the transmigration, localization, and activation of immune-inflammatory cells within the arterial wall, favoring the excessive intracellular accumulation of lipids by macrophages [11]. In venous disease, systemic oxidative inflammation and an intravascular phospholipid-*β*2GPI mediated pathway likely promote an endothelial prothrombotic dysfunction and the activation of coagulation factors. The simultaneous occurrence of these two events may have important clinical implications in cardiovascular disease.

The association between asymptomatic atherosclerosis and spontaneous venous thrombosis has been previously reported suggesting that these 2 diseases shared common risk factors [12–14]. A meta-analysis of 21 studies and 63,552 patients showed that cardiovascular risk factors were associated with venous thromboembolism (VTE) [15]. More recent reports indicate that patients with symptomatic atherosclerosis had increased risk for VTE [16] and that statins, in a dose dependent manner, decreased the incidence of VTE in 1,100 atherosclerosis patients from 22.3% to 6.3% [17]. The exact mechanism for this association remains unclear but the clinical relevance of the association has been emphasized with respect to individual screening, risk factor modification, and primary or secondary prevention of VTE. As we previously demonstrated the effect of statins on oxidative inflammation (oxLDL/*β*2GPI complexes) [10], however, the influence of statins on venous disease outcomes has not been fully evaluated. In conclusion, our results point to a possible common or overlapping risk factor for arterial and venous diseases, suggesting that high serum levels of oxLDL/*β*2GPI complexes may be used as a modifiable biomarker for evaluation and management of atherothrombotic CVD.

## 5. Conclusions

- Elevated serum levels of oxLDL/*β*2GPI complexes were associated with arterial and venous diseases.
- Male gender, dyslipidemia, and carotid disease were independent predictors of oxLDL/*β*2GPI complexes.
- Among the vascular phenotypes studied, oxLDL/*β*2GPI complexes were highest in carotid artery disease and peripheral artery disease.
- The presence of oxLDL/*β*2GPI complexes in arterial and venous diseases suggests a common oxidative mechanism among the different vascular phenotypes.

## Figures and Tables

**Table 1:** Demographic and clinical characteristics of 100 study subjects by vascular disease phenotype.

	Controls (*n* = 48)	Arterial∗ (*n* = 27)	Venous (*n* = 25)	*P* value∗∗
Age (yrs, mean ± SD)	35.4 ± 12.5	71.0 ± 11.6	54.2 ± 12.4	<0.0001
Sex (F/M)	34 F/14 M	9 F/18 M	16 F/9 M	0.005
Smoking (%)	17.0	8.3	8.0	0.422
DM (%)	0	22.2	0	0.0002
High cholesterol (%)	14.6	62.9	24.0	<0.0001
HTN (%)	8.3	55.6	24.0	<0.0001
BMI (kg/m^2^, mean ± SD)	26.3 ± 6.3	27.7 ± 5.7	30.1 ± 5.8	0.005
Fam Hx BC (%)	2.1	7.7	0	0.240
Fam Hx MI (%)	14.6	25.9	16.0	0.450
Fam Hx stroke (%)	4.2	7.4	0	0.394

^*^Arterial disease = CarAD, PAD, AAA.

∗∗Wilcoxon/Kruskal-Wallis Rank Sum or Pearson's Chi-square.

**Table 2:** Odds ratio (OR) for arterial or venous disease by oxLDL/*β*2GPI quartiles.

OxLDL/*β*2GPI quartiles∗	Arterial		Venous	
	OR (95% CI)^∧^	*P* value∗∗	OR (95% CI)^∧^	*P* value∗∗
Q2	1.25 (0.213–7.354)	0.805	6.25 (0.641–60.938)	0.085
Q3	4.44 (0.931–21.218)	0.053	18.3 (2.016–166.725)	0.002
Q4	5.42 (1.249–23.489)	0.018	10.0 (1.093–91.441)	0.020
Q1 + 2 versus Q3 + 4	4.50 (1.541–13.140)	0.004	4.10 (1.382–11.996)	0.008

^*^OxLDL/*β*2GPI quartiles: Q1 = 0–0.17; Q2 = 0.18–0.25; Q3 = 0.26–0.57; Q4 = 0.58–1.4 U/mL. The association increased significantly with oxLDL/*β*2GPI quartiles for arterial (*P* = 0.006) and venous disease (*P* = 0.015) (Cochran-Armitage Trend test).

^∧^Odds ratio (95% confidence interval).

∗∗Pearson's Chi-square against Q1.
